# (1*RS*,4*SR*)-3-Dichloro­methyl­ene-1,4-dimethyl-2-oxabicyclo­[2.2.2]oct-5-ene

**DOI:** 10.1107/S1600536808017248

**Published:** 2008-06-13

**Authors:** Andrew J. Tyrrell, George C. Feast, Jeremy Robertson

**Affiliations:** a Department of Chemistry, University of Oxford, Chemistry Research Laboratory, Mansfield Road, Oxford OX1 3TA, England

## Abstract

X-ray crystallography was used to confirm the structure of the enantio-enriched title compound, C_10_H_12_Cl_2_O, a bicylic enol ether. A bridged boat-like structure is adopted and the dichloro­methyl­ene C atom is positioned significantly removed from the core bicyclic unit. In the crystal structure, mol­ecules pack to form sheets approximately perpendicular to the *a* and *c* axes.

## Related literature

For related literature, see: Yamabe *et al.* (1996 ▶); Machiguchi *et al.* (1999 ▶); Khanjin *et al.* (1999 ▶); Ussing *et al.* (2006 ▶); Robertson & Fowler (2006 ▶).
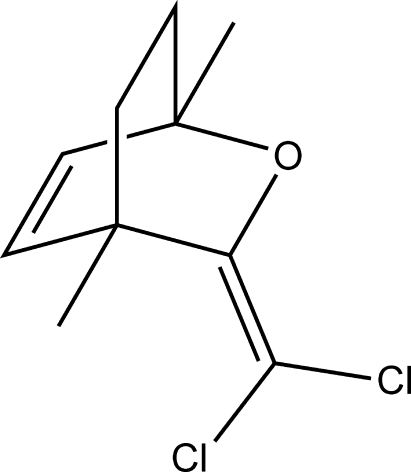


## Experimental

### 

#### Crystal data

C_10_H_12_Cl_2_O
*M* *_r_* = 219.11
Monoclinic, 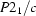

*a* = 9.3365 (1) Å
*b* = 9.6327 (2) Å
*c* = 11.4259 (2) Å
β = 92.7347 (11)°
*V* = 1026.43 (3) Å^3^
*Z* = 4
Mo *K*α radiation
μ = 0.59 mm^−1^
*T* = 150 K
0.44 × 0.32 × 0.18 mm

#### Data collection

Nonius KappaCCD diffractometer
Absorption correction: multi-scan (*DENZO*/*SCALEPACK*; Otwinowski & Minor, 1997 ▶) *T*_min_ = 0.83, *T*_max_ = 0.90
4320 measured reflections
2321 independent reflections
2094 reflections with *I* > 2σ(*I*)
*R*_int_ = 0.021

#### Refinement

*R*[*F* ^2^ > 2σ(*F* ^2^)] = 0.034
*wR*(*F* ^2^) = 0.093
*S* = 1.01
2321 reflections
118 parameters
2 restraints
H-atom parameters constrained
Δρ_max_ = 0.36 e Å^−3^
Δρ_min_ = −0.38 e Å^−3^

### 

Data collection: *COLLECT* (Nonius, 2001 ▶); cell refinement: *DENZO*/*SCALEPACK* (Otwinowski & Minor, 1997 ▶); data reduction: Görbitz (1999 ▶) and *DENZO*/*SCALEPACK*; program(s) used to solve structure: *SIR92* (Altomare *et al.*, 1994 ▶); program(s) used to refine structure: *CRYSTALS* (Betteridge *et al.*, 2003 ▶); molecular graphics: *CAMERON* (Watkin *et al.*, 1996 ▶); software used to prepare material for publication: *CRYSTALS*.

## Supplementary Material

Crystal structure: contains datablocks I, global. DOI: 10.1107/S1600536808017248/lh2637sup1.cif

Structure factors: contains datablocks I. DOI: 10.1107/S1600536808017248/lh2637Isup2.hkl

Additional supplementary materials:  crystallographic information; 3D view; checkCIF report

## Figures and Tables

**Fig. 1.**
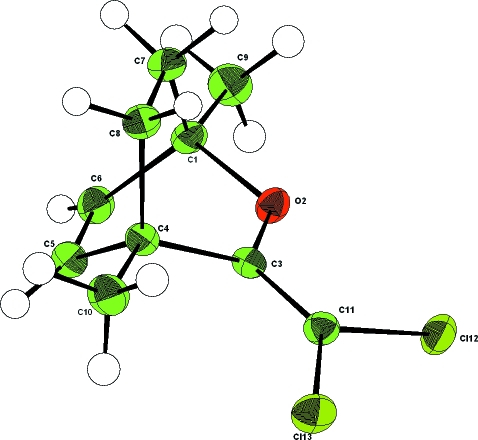
The title compound with displacement ellipsoids drawn at the 50% probability level. H atoms are shown as spheres of arbitary radius.

**Fig. 2.**
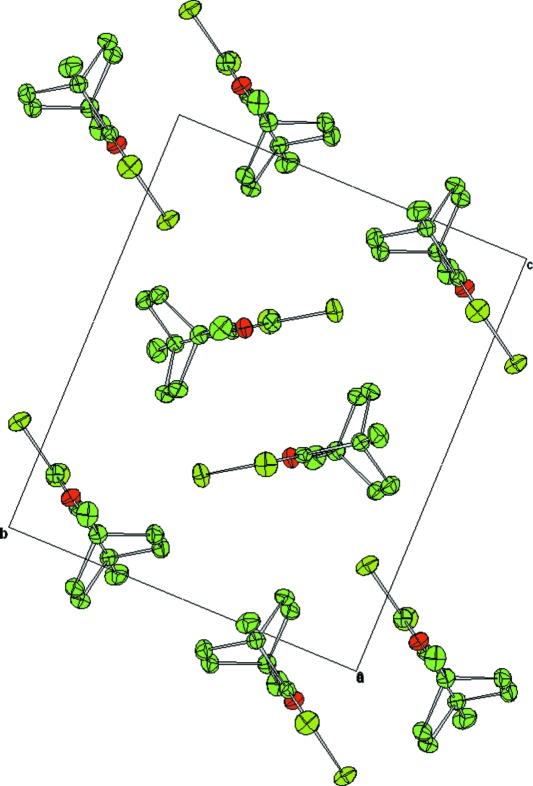
The title compound viewed along the a-axis with H atoms omitted.

**Fig. 3.**
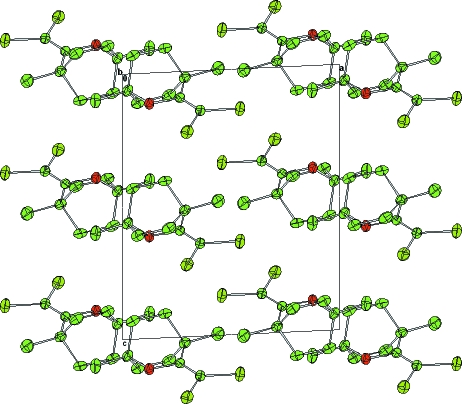
The title compound viewed along the b-axis with H atoms omitted.

## supplementary crystallographic information

### Comment

The reaction between dienes and ketenes to produce cyclobutanones was long considered to be a textbook example of a [2 + 2] cycloaddition that could be understood in terms of a π2_s_ + π2_a_Woodward–Hoffmann formalism. More recently, evidence has been presented for a stepwise hetero-Diels–Alder/Claisen rearrangement pathway (Yamabe *et al.*, 1996) and it was reported that the periselectivity of these cycloadditions is responsive to the nature of the diene (Machiguchi *et al.*, 1999). The situation is, however, more complex and a combined theoretical and experimental study of the reaction of cyclopentadiene with either dichloro- or diphenylketene revealed that both [4 + 2] and [2 + 2] adducts may be produced directly through parallel reaction pathways traversing a bifurcating energy surface (Ussing *et al.* 2006). Our studies sought to address certain mechanistic aspects of the Claisen rearrangement of bicyclic enol ethers structurally analogous to those produced in diene/ketene [4 + 2] cycloadditions (Robertson & Fowler, 2006); within this study, although crystals were obtained as a racemate, the title compound was prepared in an enantioenriched form in order to determine if access to non-racemic cyclobutanones could be achieved.

The relationship between computed distances of reacting termini and activation energies has been discussed for structurally similar Claisen precursors in the context of the mechanism of chorismate mutase (Khanjin *et al.*, 1999). The molecular stucture (Fig. 1) shows the dichloromethylene carbon to be significantly removed from the carbon at C5 (3.5523 Å) and yet the title compound can be induced to undergo the Claisen rearrangement under mild thermal conditions to yield (1*RS*, 6*SR*)-8,8-dichloro-3,6-dimethylbicyclo[4.2.0]oct-3-en-7-one. Also of note are the sheets of molecules which form approximatedly perpendicular to the *a*- and *c*-axes as shown in Fig. 2 and Fig. 3.

### Experimental

The title compound was crystallized by concentration of a sample dissolved in petroleum ether. [α]_D_^25^-36.1 (CHCl_3_, *c *= 1.0).

### Refinement

Changes in illuminated volume were kept to a minimum, and were taken into account (Görbitz, 1999) by the multi-scan inter-frame scaling (*DENZO*/*SCALEPACK*, Otwinowski & Minor, 1997).

The H atoms were all located in a difference map, but those attached to carbon atoms were repositioned geometrically. The H atoms were initially refined with soft restraints on the bond lengths and angles to regularize their geometry (C—H in the range 0.93–0.98) and *U*_iso_(H) (in the range 1.2–1.5 times *U*_eq_ of the parent atom), after which the positions were refined with riding constraints.

### Crystal data

**Table:**

C_10_H_12_Cl_2_O_1_	*F*_000_ = 456
*M**_r_* = 219.11	*D*_x_ = 1.418 Mg m^−^^3^
Monoclinic, *P*2_1_/*c*	Mo *K*α radiation λ = 0.71073 Å
Hall symbol: -P 2ybc	Cell parameters from 15784 reflections
*a* = 9.3365 (1) Å	θ = 5–27º
*b* = 9.6327 (2) Å	µ = 0.59 mm^−^^1^
*c* = 11.4259 (2) Å	*T* = 150 K
β = 92.7347 (11)º	Prism, colourless
*V* = 1026.43 (3) Å^3^	0.44 × 0.32 × 0.18 mm
*Z* = 4	

### Data collection

**Table:**

Nonius KappaCCD diffractometer	2094 reflections with *I* > 2σ(*I*)
Monochromator: graphite	*R*_int_ = 0.021
*T* = 150 K	θ_max_ = 27.4º
ω scans	θ_min_ = 5.1º
Absorption correction: multi-scan(DENZO/SCALEPACK; Otwinowski & Minor, 1997)	*h* = −12→12
*T*_min_ = 0.83, *T*_max_ = 0.90	*k* = −12→12
4320 measured reflections	*l* = −14→14
2321 independent reflections	

### Refinement

**Table:**

Refinement on *F*^2^	Primary atom site location: structure-invariant direct methods
Least-squares matrix: full	Hydrogen site location: inferred from neighbouring sites
*R*[*F*^2^ > 2σ(*F*^2^)] = 0.034	H-atom parameters constrained
*wR*(*F*^2^) = 0.093	Method = Modified Sheldrick *w* = 1/[σ^2^(*F*^2^) + (0.05*P*)^2^ + 0.71*P*], where *P* = [max(*F*_o_^2^,0) + 2*F*_c_^2^]/3
*S* = 1.01	(Δ/σ)_max_ = 0.001
2321 reflections	Δρ_max_ = 0.36 e Å^−^^3^
118 parameters	Δρ_min_ = −0.38 e Å^−^^3^
2 restraints	Extinction correction: None

### Fractional atomic coordinates and isotropic or equivalent isotropic displacement parameters (Å^2^)

**Table:**

	*x*	*y*	*z*	*U*_iso_*/*U*_eq_	
C1	0.02322 (16)	0.72676 (17)	0.56170 (14)	0.0234	
O2	0.12271 (11)	0.62312 (12)	0.61475 (10)	0.0217	
C3	0.26458 (15)	0.64956 (16)	0.59821 (13)	0.0192	
C4	0.28716 (17)	0.77747 (17)	0.52291 (14)	0.0230	
C5	0.21390 (19)	0.89526 (18)	0.58719 (15)	0.0293	
C6	0.07027 (19)	0.86685 (17)	0.60962 (15)	0.0274	
C7	0.04601 (18)	0.72396 (18)	0.43089 (14)	0.0280	
C8	0.19575 (18)	0.75082 (18)	0.40893 (14)	0.0267	
C9	−0.12456 (17)	0.6806 (2)	0.59357 (17)	0.0326	
C10	0.44060 (19)	0.8143 (2)	0.49372 (17)	0.0339	
C11	0.35800 (16)	0.56064 (17)	0.64983 (14)	0.0215	
Cl12	0.29697 (4)	0.42007 (4)	0.72783 (4)	0.0295	
Cl13	0.54194 (4)	0.56859 (5)	0.64989 (4)	0.0320	
H51	0.2631	0.9775	0.6123	0.0403*	
H61	0.0073	0.9265	0.6509	0.0362*	
H71	−0.0214	0.7925	0.3907	0.0397*	
H72	0.0158	0.6344	0.3998	0.0386*	
H81	0.1990	0.8293	0.3587	0.0379*	
H82	0.2347	0.6712	0.3675	0.0381*	
H91	−0.1917	0.7492	0.5652	0.0477*	
H92	−0.1261	0.6724	0.6804	0.0498*	
H93	−0.1529	0.5941	0.5590	0.0483*	
H101	0.4319	0.8976	0.4440	0.0529*	
H102	0.5032	0.8374	0.5608	0.0543*	
H103	0.4864	0.7409	0.4472	0.0531*	

### Atomic displacement parameters (Å^2^)

**Table:**

	*U*^11^	*U*^22^	*U*^33^	*U*^12^	*U*^13^	*U*^23^
C1	0.0213 (7)	0.0229 (7)	0.0254 (8)	0.0045 (6)	−0.0044 (6)	−0.0009 (6)
O2	0.0175 (5)	0.0225 (6)	0.0251 (5)	0.0016 (4)	0.0005 (4)	0.0041 (4)
C3	0.0188 (7)	0.0205 (7)	0.0183 (7)	−0.0023 (5)	0.0005 (5)	−0.0019 (6)
C4	0.0257 (7)	0.0220 (7)	0.0209 (7)	−0.0043 (6)	−0.0022 (5)	0.0018 (6)
C5	0.0373 (8)	0.0221 (8)	0.0275 (8)	0.0009 (7)	−0.0073 (7)	−0.0031 (7)
C6	0.0339 (8)	0.0229 (8)	0.0251 (8)	0.0055 (7)	−0.0030 (6)	−0.0032 (6)
C7	0.0347 (8)	0.0264 (8)	0.0223 (8)	0.0014 (7)	−0.0067 (6)	−0.0013 (6)
C8	0.0352 (8)	0.0268 (8)	0.0178 (7)	−0.0025 (7)	−0.0026 (6)	0.0010 (6)
C9	0.0212 (8)	0.0328 (9)	0.0435 (10)	0.0028 (7)	−0.0005 (7)	−0.0031 (8)
C10	0.0309 (9)	0.0345 (10)	0.0360 (9)	−0.0126 (7)	−0.0013 (7)	0.0072 (8)
C11	0.0189 (7)	0.0234 (7)	0.0222 (7)	−0.0011 (6)	0.0001 (5)	0.0004 (6)
Cl12	0.0292 (2)	0.0261 (2)	0.0329 (2)	−0.00012 (15)	−0.00257 (16)	0.01006 (16)
Cl13	0.0183 (2)	0.0372 (3)	0.0401 (3)	0.00107 (15)	−0.00273 (16)	0.00256 (18)

### Geometric parameters (Å, °)

**Table:**

C1—O2	1.4741 (18)	C7—C8	1.455 (2)
C1—C6	1.514 (2)	C7—H71	1.008
C1—C7	1.520 (2)	C7—H72	0.970
C1—C9	1.511 (2)	C8—H81	0.950
O2—C3	1.3707 (17)	C8—H82	0.980
C3—C4	1.523 (2)	C9—H91	0.957
C3—C11	1.339 (2)	C9—H92	0.996
C4—C5	1.531 (2)	C9—H93	0.954
C4—C8	1.544 (2)	C10—H101	0.985
C4—C10	1.528 (2)	C10—H102	0.968
C5—C6	1.404 (3)	C10—H103	0.993
C5—H51	0.953	C11—Cl12	1.7324 (16)
C6—H61	0.962	C11—Cl13	1.7190 (15)
			
O2—C1—C6	106.78 (12)	C1—C7—H72	108.9
O2—C1—C7	106.10 (12)	C8—C7—H72	111.1
C6—C1—C7	108.65 (14)	H71—C7—H72	104.6
O2—C1—C9	105.44 (13)	C4—C8—C7	112.39 (13)
C6—C1—C9	115.34 (14)	C4—C8—H81	110.2
C7—C1—C9	113.84 (14)	C7—C8—H81	107.7
C1—O2—C3	114.30 (12)	C4—C8—H82	109.5
O2—C3—C4	112.89 (12)	C7—C8—H82	109.0
O2—C3—C11	115.71 (13)	H81—C8—H82	107.9
C4—C3—C11	131.40 (14)	C1—C9—H91	107.8
C3—C4—C5	104.57 (13)	C1—C9—H92	108.7
C3—C4—C8	104.84 (12)	H91—C9—H92	110.5
C5—C4—C8	106.63 (13)	C1—C9—H93	113.3
C3—C4—C10	117.88 (14)	H91—C9—H93	107.4
C5—C4—C10	112.19 (14)	H92—C9—H93	109.1
C8—C4—C10	109.92 (13)	C4—C10—H101	105.3
C4—C5—C6	113.31 (14)	C4—C10—H102	114.7
C4—C5—H51	122.7	H101—C10—H102	107.4
C6—C5—H51	123.9	C4—C10—H103	112.5
C1—C6—C5	111.77 (14)	H101—C10—H103	107.3
C1—C6—H61	122.4	H102—C10—H103	109.2
C5—C6—H61	125.8	C3—C11—Cl12	120.21 (12)
C1—C7—C8	110.31 (13)	C3—C11—Cl13	126.97 (12)
C1—C7—H71	108.8	Cl12—C11—Cl13	112.82 (9)
C8—C7—H71	112.9		

## References

[bb1] Altomare, A., Cascarano, G., Giacovazzo, C., Guagliardi, A., Burla, M. C., Polidori, G. & Camalli, M. (1994). *J. Appl. Cryst.* **27**, 435.

[bb2] Betteridge, P. W., Carruthers, J. R., Cooper, R. I., Prout, K. & Watkin, D. J. (2003). *J. Appl. Cryst.* **36**, 1487.

[bb11] Görbitz, C. H. (1999). *Acta Cryst* B**55**, 1090–1098. 10.1107/s0108768199008721 10927450

[bb3] Khanjin, N. A., Snyder, J. P. & Menger, F. M. (1999). *J. Am. Chem. Soc.* **121**, 11831–11846.

[bb4] Machiguchi, T., Hasegawa, T., Ishiwata, A., Terashima, S., Yamabe, S. & Minato, T. (1999). *J. Am. Chem. Soc.* **121**, 4771–4786.

[bb5] Nonius (2001). *COLLECT* Nonius BV, Delft, The Netherlands.

[bb6] Otwinowski, Z. & Minor, W. (1997). *Methods in Enzymology*, Vol. 276, *Macromolecular Crystallography*, Part A, edited by C. W. Carter Jr & R. M. Sweet, pp. 307–326. New York: Academic Press.

[bb7] Robertson, J. & Fowler, T. (2006). *Org. Biomol. Chem.* **4**, 4307–4318. 10.1039/b611311g 17102876

[bb8] Ussing, B. R., Hang, C. & Singleton, D. A. (2006). *J. Am. Chem. Soc.* **128**, 7594–7607. 10.1021/ja0606024 PMC2453781 16756316

[bb9] Watkin, D. J., Prout, C. K. & Pearce, L. J. (1996). *CAMERON* Chemical Crystallography Laboratory, Oxford, UK.

[bb10] Yamabe, S., Dai, T., Minato, T., Machiguchi, T. & Hasegawa, T. (1996). *J. Am. Chem. Soc.* **118**, 6518–6519.

